# Indole-3-propionic acid alleviates sepsis-associated acute liver injury by activating pregnane X receptor

**DOI:** 10.1186/s10020-023-00658-x

**Published:** 2023-05-19

**Authors:** Shuang Wang, Liangzhi Xiong, Zhihua Ruan, Xiaofang Gong, Yanrong Luo, Chengyi Wu, Yu Wang, Hui Shang, Jingyi Chen

**Affiliations:** 1 Department of Anesthesiology, Taihe Hospital, Hubei University of Medicine, Shiyan, 442000 Hubei China; 2 Physical examination center, Shiyan Hospital of Integrated Traditional and Western Medicine, Shiyan, 442000 Hubei China; 3 Department of Orthopaedic, Taihe Hospital, Hubei University of Medicine, Shiyan, 442000 Hubei China

**Keywords:** Sepsis, Acute liver injury, Indole-3-propionic acid, Pregnane X receptor, Gut microbiota

## Abstract

**Background:**

The morbidity and mortality of sepsis are extremely high, which is a major problem plaguing human health. However, current drugs and measures for the prevention and treatment of sepsis have little effect. Sepsis-associated acute liver injury (SALI) is an independent risk factor for sepsis, which seriously affects the prognosis of sepsis. Studies have found that gut microbiota is closely related to SALI, and indole-3-propionic Acid (IPA) can activate Pregnane X receptor (PXR). However, the role of IPA and PXR in SALI has not been reported.

**Methods:**

This study aimed to explore the association between IPA and SALI. The clinical data of SALI patients were collected and IPA level in feces was detected. The sepsis model was established in wild-type mice and PXR knockout mice to investigate the role of IPA and PXR signaling in SALI.

**Results:**

We showed that the level of IPA in patients’ feces is closely related to SALI, and the level of IPA in feces has a good ability to identify and diagnose SALI. IPA pretreatment significantly attenuated septic injury and SALI in wild-type mice, but not found in knockout PXR gene mice.

**Conclusions:**

IPA alleviates SALI by activating PXR, which reveals a new mechanism of SALI, and provides potentially effective drugs and targets for the prevention of SALI.

**Supplementary Information:**

The online version contains supplementary material available at 10.1186/s10020-023-00658-x.

## Introduction

Sepsis is a life-threatening organ dysfunction caused by the body’s maladjusted response to infection (Zheng et al. 2021). At present, the morbidity and mortality of sepsis are high, and although many advances have been made in clinical sepsis management and improved outcomes, including adequate source control, resuscitation, and early use of broad-spectrum antibiotics, the specific, targeted therapies are still lacking (Deng et al. 2022a, b, c; Shi et al. 2021; Sun et al. 2021). Therefore, it is of great scientific value and clinical significance to explore the prevention and treatment measures of sepsis. The liver is an important organ in the human body that regulates immune defense by eliminating bacteria, producing acute-phase proteins and cytokines, and generating adaptive inflammatory responses (Mayneris-Perxachs et al. 2021; Rao et al. 2021; Gao et al. 2021; Ghodsian et al. 2021; Ahmed et al. 2021; Wang et al. 2021a, b; Bulfoni et al. 2021). Liver dysfunction seriously affects the prognosis of patients with sepsis and is an independent predictor of ICU mortality (Woźnica et al. 2018; Sun et al. 2020). Therefore, early detection and timely intervention of patients with sepsis-associated acute liver injury (SALI) are essential.

There is a close interaction between the gut and liver (Ahmed et al. 2021; Wang et al. 2021a, b, 2022; Li et al. 2022; Sangineto et al. 2022; Xiang et al. 2022; Khan et al. 2021). Disruption of the intestinal barrier and dysregulation of the intestinal microbiome during sepsis result in migration of intestinal pathogen-associated molecular patterns and injury-associated molecular patterns into the liver and systemic circulation. The liver is critical for regulating immune defense during systemic infection through mechanisms such as bacterial clearance, lipopolysaccharide detoxification, cytokine and acute phase protein release, and inflammatory metabolism regulation. Impaired pathogen clearance and disrupted liver metabolism can lead to further damage to the intestinal barrier and increased disruption of intestinal microbiome composition and diversity when the liver undergoes an inappropriate immune response or excessive inflammation. Therefore, the interaction between the gut and the liver is a potential therapeutic target (Deng et al. 2021a, b, 2022a, b, c). The studies have found that sepsis coincides with perturbations in the composition of intestinal microbiota (Zhang et al. 2020; Agudelo-Ochoa et al. 2020; Adelman et al. 2020; Miller et al. 2021; Deng et al. 2023). Intestinal flora affects the occurrence and development of sepsis by acting on the body’s immune response, intestinal barrier function and enterohepatic circulation (Haak and Wiersinga 2017). IPA treatment inhibited intestinal flora disturbance in mice with sepsis (Fang et al. 2022a, b), increased the phagocytosis of macrophages against bacteria (Huang et al. 2022), inhibited neuroinflammation in sepsis-associated encephalopathy (Fang et al. 2022a, b). However, the role and potential mechanism of IPA in SALI have not been reported.

Pregnane X receptor (PXR) is one of the nuclear receptor superfamily receptors. Its main function is to regulate the metabolism of heterologous substances, such as sugars and lipids, and participate in the maintenance of normal liver function (Barretto et al. 2021; Jiang et al. 2019). It has been found that sepsis inhibits PXR receptor expression (Sachdeva et al. 2003) and PXR-signaling is influenced by microbiota-derived IPA in the intestine (Flannigan et al. 2022). These studies suggest that IPA may attenuate SALI by activating PXR. However, the association between IPA and SALI, IPA and PXR, and PXR and SALI has not been reported in sepsis.

In this study, we investigated the role of IPA in SALI and the role of PXR signaling in IPA attenuating SALI. We aimed to reveal the new mechanism of SALI and provide potential therapeutic drugs for the prevention and treatment of SALI.

## Materials and methods

### Patients

This study involving human participants, human material and human data was approved by the Ethics Committee of Hubei University of Medicine and Taihe hospital, and the study was performed in accordance with the code of ethics of the world medical association (Declaration of Helsinki). The written informed consent was obtained from all enrolled patients. Sepsis and septic shock were defined according to the third edition of the International Definition of Sepsis and Septic shock (Sepsis-3). Patients in the “SALI” group were eligible if their blood laboratory results met at least one of the following four conditions: (i) serum total bilirubin (tbil) ≥ 3.0 mg/dL; (ii) aspartate aminotransferase (AST) ≥ 41 IU/L; Iron (iii) alanine amino trans enzyme (ALT) for 41 IU/L or higher; (iv) γ-glutamyl transpeptidase (GGT) of 51 IU/L or higher (Kobashi et al. 2013). Patients diagnosed with sepsis but not meeting the SALI diagnostic criteria were classified as the “sepsis non-acute liver injury” group (SNALI). Patients diagnosed with sepsis within one day of ICU admission were enrolled in the study, stool samples and blood samples were collected from the patient on the same day.

### Mice

For this study, 6–8 week-old male C57BL/6J mice were purchased from the animal center of Hubei University of Medicine (Shiyan, China). All mouse experimental procedures were approved by the Institutional Animal Care and Use Committee of Hubei University of Medicine. All mice were housed under constant temperature and humidity, with a 12-h light-dark cycle, had free access to food and water, and fasted overnight before the experiment.

### Mouse sepsis model and mouse sepsis injury score (MSS)

The mouse sepsis model by cecal ligation and puncture was constructed as described previously (Rittirsch et al. 2009). Briefly, After 3 days of adaptive feeding, mice were fasted for 12 h before establishing CLP model. After anesthesia, mice were skin-prepared, sterilized, and fixed. The midline of the skin was cut longitudinally with a scalpel, and the cecum was located and separated and removed. Ligate 1/3 of the cecum with No. 3 thread. The ligation end was perforated with an 18-gauge needle, a small amount of feces was squeezed out, and the peritoneum and skin were sutured discontinuously with a 4-gauge silk thread. After the suture closed the peritoneum, fascia, and abdominal muscle tissue, preheated saline was injected subcutaneously (37 °C; 5 ml per 100 g body weight).

MSS score was used to evaluate the severity of sepsis in mice. MSS has been described previously (Shrum et al. 2014); In brief, seven observation measures, including appearance, level of consciousness, activity, response to stimuli, eyes, respiratory rate, and respiratory quality, were scored from 0 to 4 out of 28 points each. A higher score indicates a more severe injury.

### Fecal microbiota transplantation

To observe the role of gut microbiota in SALI, fecal microbiota transplantation experiments were performed according to previously described methods (Deng et al. 2023). In brief, male C57BL/6J mice of 6 to 8 weeks old purchased from the same batch were housed together in large cages. Antibiotics (ABX)(Vancomycin, 100 mg/kg; Neomycin sulfate 200 mg/kg; Metronidazole 200 mg/kg; Ampicillin 200 mg/kg) was administrated intragastric for 1 week to consume intestinal flora (pseudosterile mice). Fecal grafts come from a collection of feces. The feces of the donor patients in the SALI and SNALI groups were resuspended in 0.125 g/mL PBS and refrigerated at -80℃. Subsequently, 100 mL fecal fluid was intragastrically administered to the corresponding group of pseudosterile mice (recipient mice) for 7 consecutive days. The pseudosterile mice receiving the SALI and SNALI feces were called the SALI feces group and SNALI feces group, respectively.

### RNA extraction and RT–PCR

RNA was extracted with TRIzol reagent (Invitrogen, New York, USA). Real-time PCR was performed using the ABI Q5 Real-Time PCR System (Applied Biosystems, Foster City, CA, USA) with the SYBR Green detection protocol (TOYOBO, Tokyo, Japan). The expression of target genes in mice was normalized against that of the housekeeping gene 18 S using the 2^-ΔΔCT^ method. The quantitative RT-PCR primer sequence: 18 S, Forward primer (5’–3’) CGATCCGAGGGCCTCACTA, Reverse primer (5’–3’) AGTCCCTGCCCTTTGTACACA; PXR, Forward primer (5’–3’) GATGGAGGTCTTCAAATCTGCC, Reverse primer (5’–3’) CAGCCGGACATTGCGTTTC; ZO-1, Forward primer (5’–3’) AGAGACAAGATGTCCGCCAG, Reverse primer (5’–3’) TGCAATTCCAAATCCAAACC; Occludin, Forward primer (5’–3’) CATTTATGATGAACAGCCCC, Reverse primer (5’–3’) GGACTGTCAACTCTTTCCGC.

### Quantification of IPA

The cecal contents of mice were collected 12 h after CLP model was established. Preparation and extraction of fecal supernatant samples as previously mentioned (Xue et al. 2022). IPA was determined by ultra-high performance liquid chromatography (UPLC, Agilent) coupled mass spectrometry (MS, Agilent). The injection volume was 5µL. The flow rate was set to 200 µL/min. Mass spectrometer work in electrospray ionization (ESI) mode, capillary 2.24 kV voltage, temperature desolventizing line to -450 ℃, hot piece of temperature to -200 ℃, cone gas for the − 1.5 L/min, nitrogen − 12 L/min. The calibration curve of IPA was between 1 ng/mL and 250 ng/mL using ESTD. All analyses were linear. All the standard products come from Sigma-Aldrich and the solvent comes from Merck.

### Hematoxylin and eosin (H&E) staining and detection of liver injury

Liver tissue samples were collected and fixed in 4% paraformaldehyde. Then, the samples were embedded in paraffin, 5-µm thick sections were stained with H&E according to standard methods. Images were captured at 200x with an Olympus fluorescence microscope (Olympus, Tokyo, Japan). The injury of liver sections was scored by blinded technicians (Suzuki et al. 1993). An alanine aminotransferase (ALT) assay kit (C009-2-1, Nanjing Jiancheng Bioengineering Institute) and aspartate aminotransferase assay kit (C010-2-1, Nanjing Jiancheng Bioengineering Institute) were used to detect ALT and AST levels in plasma to reflect liver damage.

### Statistical analysis

The data were analyzed using GraphPad Prism software (version 7.0). Categorical variables were expressed as frequency and percentage (n, %), and comparison between groups was performed using the chi-square test or Fisher’s exact test. Continuous variables with normal distribution were expressed as mean ± standard deviation (mean ± SD), and comparison between groups was performed using the ANOVA and post-hoc test. The measurement data of non-normal distribution were expressed as median (interquartile range, IQR), and the rank sum test was used for comparison between groups. Mice survival was assessed by Chi-square test. A value of *p* < 0.05 was considered significant.

## Results

### Fecal levels of IPA, a metabolite of the gut microbiota, were strongly correlated with SALI

**Fig. 1:**
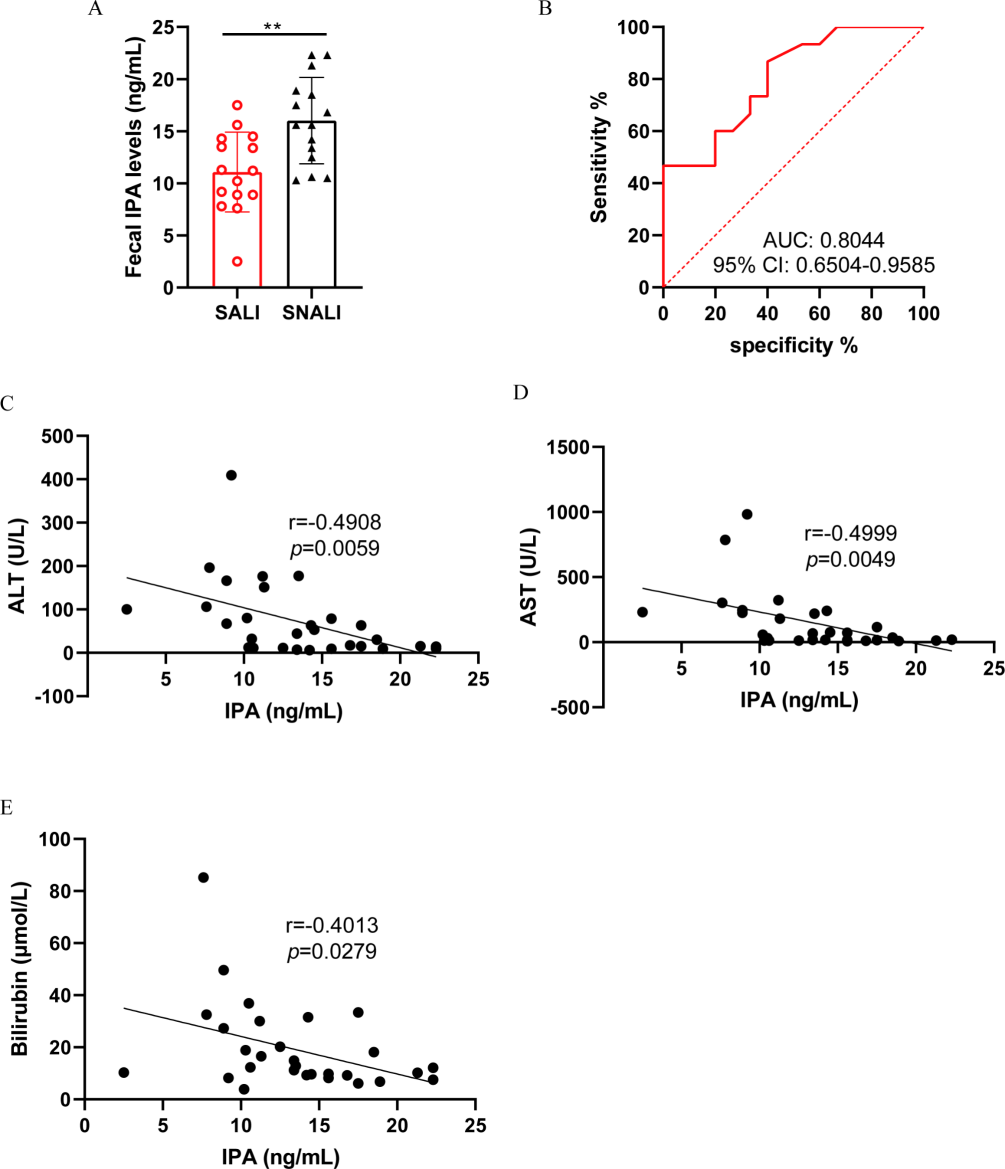
**The lower IPA levels in feces correlate with more severe SALI.** (**A**) The fecal IPA levels in patients (n = 15). (**B**) The receiver operating characteristic curves were used to identify SALI (n = 15). (**C**-**E**) The correlation analysis was performed between the level of IPA in stool and the level of ALT (**C**), AST (**D**), and bilirubin (**E**) on the day the patient was diagnosed with sepsis. IPA, Indole-3-propionic acid; SALI, sepsis-associated acute liver injury. The results are expressed as the mean ± SD, “*” indicates *p* < 0.05, “**” indicates *p* < 0.01, “***” indicates *p* < 0.001, *p* were determined by ANOVA and post-hoc test or by spearman analysis

To observe the association between IPA and patients with sepsis-associated acute liver injury (SALI), we included 15 patients with SALI and 15 patients with sepsis but without acute liver injury (SNALI). As shown in Table 1, there were no significant differences in age, gender and initial infection site between the two groups. Compared with the SNALI group, patients in the SALI group had higher mortality, longer length of ICU stay, and higher SOFA score on the day of sepsis diagnosis. Among the laboratory tests, the levels of creatinine, bilirubin, ALT and AST were significantly higher in the SALI group on the day of sepsis diagnosis. Furthermore, the level of fecal IPA was significantly lower in the SALI group than that in the SNALI group (Fig. 1A). The results of receiver operating characteristic curve showed that the level of IPA in feces on the day of sepsis diagnosis had a good potential to identify and distinguish SALI (Fig. 1B). In addition, the level of IPA in the patient’s stool was significantly correlated with the level of ALT (Fig. 1C), AST (Fig. 1D), and bilirubin (Fig. 1E) on the day the patient was diagnosed with sepsis. All these data suggested fecal levels of IPA, a metabolite of the gut microbiota, were strongly correlated with SALI.

**Table 1:** Patient clinical characteristics

Characteristics	SALI	SNALI	*P*
Number of patients included (n)	15	15	
Age (years)	64.7 (39–86)	58.6 (23–79)	0.294
Males (n)	12 (80.00)	9 (60.0 )	0.24
SOFA	11 (8–14)	5 (4–6)	0.005
**Laboratory**			
BUN (mg/dL)	14.43 ± 2.94	14.16 (-1.16-29.49)	0.971
Cr (umol/L)	219.2 (143.2-295.2)	92.67 (60.3-125.1)	0.004
ALT (U/L)	128.6 (77.1-180.1)	13.9 (9.6–18.1)	0.0003
AST (U/L)	274.7 (128.2-428.1)	16.7 (12.3–21.1)	0.002
Bilirubin (µmol/L)	25.3 (13.9–36.8)	15.2 (8.8–17.5)	0.047
**Clinical Outcomes**			
Mortality within 28 days (n/%)	6 (40.00)	1 (6.67)	0.031
hospitalization time (d)	17.3 ± 6.93	11.2 ± 3.58	0.024

SALI, sepsis-associated acute liver injury; SNALI, sepsis without acute liver injury; SOFA, sequential organ failure assessment; BUN, blood urea nitrogen; ALT, Alanine transaminase; AST, Aspartate transamina

### Gut microbiota affects SALI

To observe changes in fecal IPA levels after antibiotic clearance of intestinal bacteria or CLP models, mice were gavaged with antibiotics (ABX) for 1 week to deplete gut microbiota (ABX group) or CLP model was established after 1 week of PBS intragastric administration (CLP group) (Fig. S1A). The results of targeted metabolomics showed that the level of IPA in the feces of mice was significantly reduced after ABX deletion of gut microbiota or CLP (Fig. S1B). These results suggest that IPA is a metabolite of gut microbiota.

**Fig. 2:**
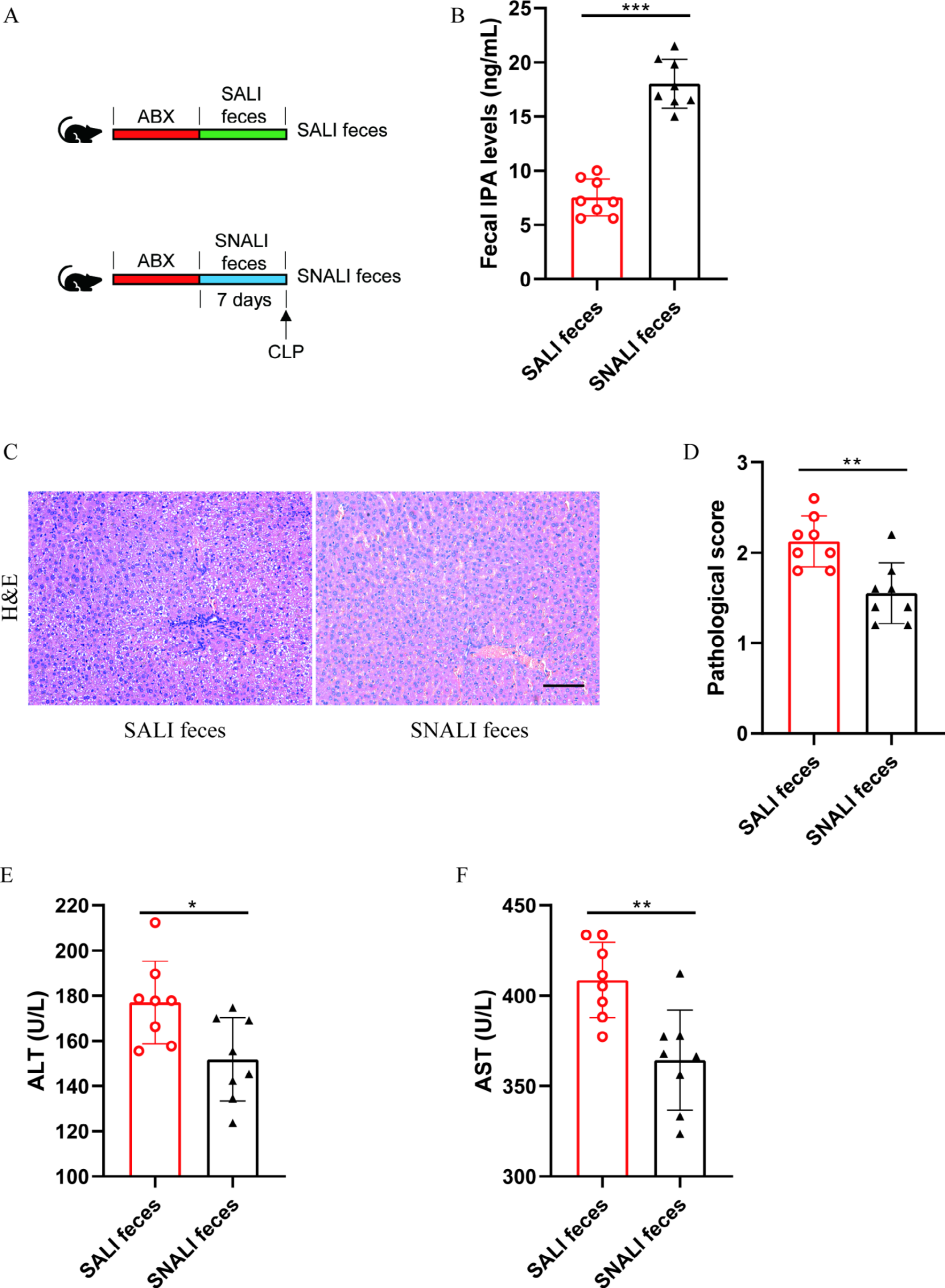
**Gut microbiota affects SALI.** (**A**) The schematic diagram of experimental grouping of mice. (**B**) The fecal IPA levels in mice (n = 8). (**C**-**D**) The H&E staining (**C**) and pathological scoring (**D**) of liver tissue sections (n = 8), the scale bar is 100 μm. (**E**) The plasma ALT levels in mice (n = 8). (**F**) The plasma AST levels in mice (n = 8). The results are expressed as the mean ± SD, “*” indicates *p* < 0.05, “**” indicates *p* < 0.01, “***” indicates *p* < 0.001, *p* were determined by ANOVA and post-hoc test

To observe the role of gut microbiota in SALI, the feces from the SALI group or SNALI group were transplanted into pseudosterile mice to establish a model of sepsis (Fig. 2A). The IPA level in the feces of SALI feces treated mice was significantly lower than that of SNALI feces treated mice before CLP model establishment (Fig. 2B), which suggested that fecal microbiota transplantation was successful. After the establishment of sepsis, the SALI feces group had significantly higher liver tissue H&E damage (Fig. 2C-D) and plasma ALT (Fig. 2E) and AST (Fig. 2F) levels than the SNALI feces group. These results suggested that gut microbiota is an important factor affecting SALI, which may be related to IPA levels.

### IPA attenuates septic injury in mice

**Fig. 3:**
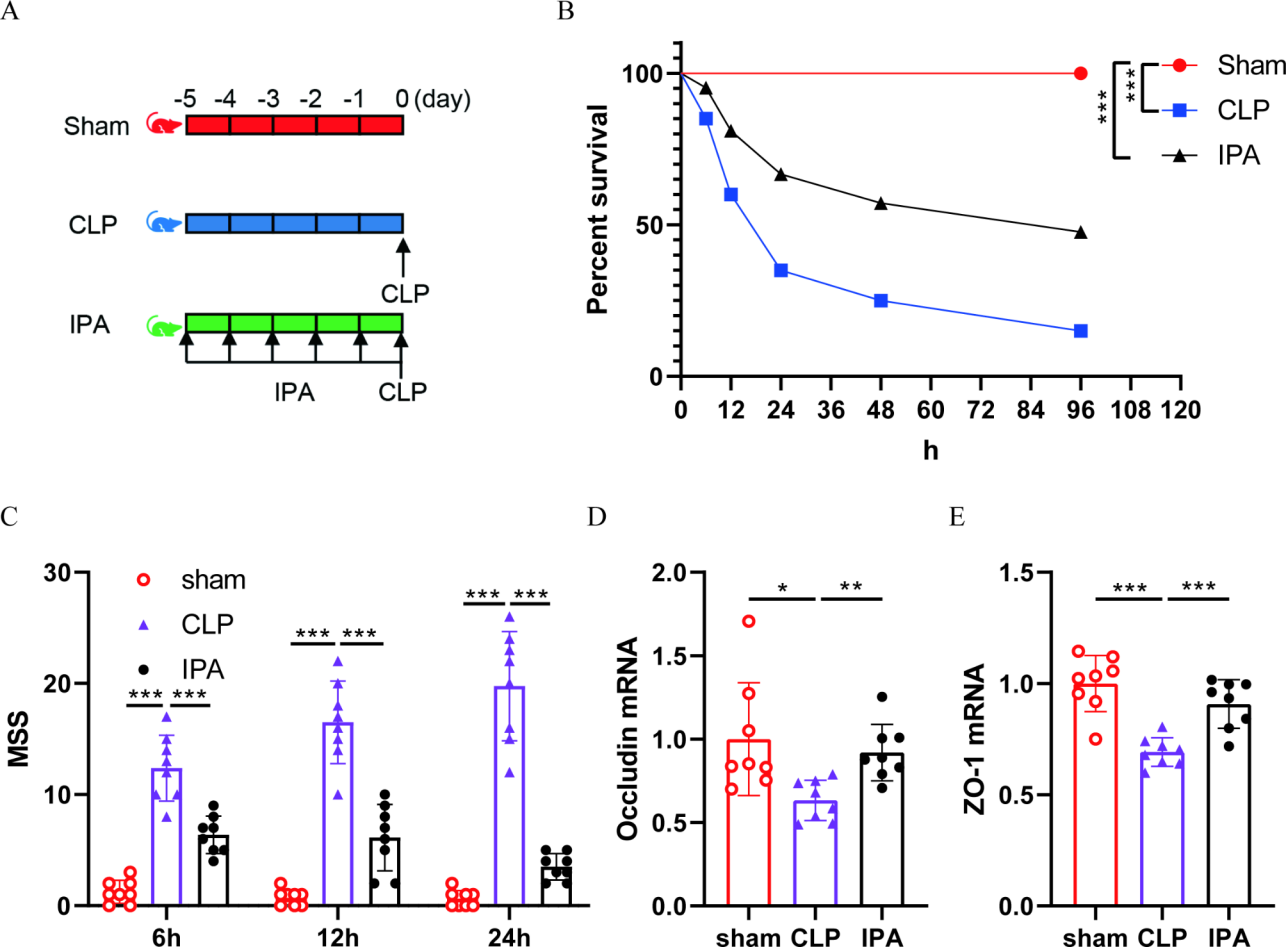
**IPA attenuates septic injury in mice.** (**A**) The schematic diagram of experimental grouping of mice. (**B**) The survival rate of mice (n = 20). (**C**) The mice sepsis injury score (MSS). (**D**-**E**) Relative mRNA levels of tight junction marker Occludin (**D**) and ZO-1 (**E**) in intestinal tissue. The results are expressed as the mean ± SD, “*” indicates *p* < 0.05, “**” indicates *p* < 0.01, “*** or ^###^” indicates *p* < 0.001, *p* were determined by Chi-square test **(B**) or ANOVA and post-hoc test (**C**-**E**).

To observe the effect of IPA in septic mice, mice were given IPA (20 mg/kg) or vehicle by gavage daily for 5 consecutive days before the establishment of CLP model (Fig. 3A). The results of mouse survival experiments showed that IPA significantly inhibited the high mortality rate of CLP model mice (Fig. 3B). In addition, the MSS scores of IPA treated mice were significantly lower than those of CLP mice at 6 h, 12 and 24 h after operation (Fig. 3C). These results suggested that IPA has a significant effect on reducing septic injury.

The intestinal barrier effectively prevents harmful substances such as bacteria and toxins from entering other tissues, organs or the blood circulation. IPA treatment significantly promoted the expression of tight junction markers Occludin (Fig. 3D) and ZO-1 (Fig. 3E) in intestinal tissue of sepsis mice, which suggested that IPA treatment maintained intestinal barrier homeostasis during sepsis.

### IPA alleviates liver injury in septic mice

**Fig. 4:**
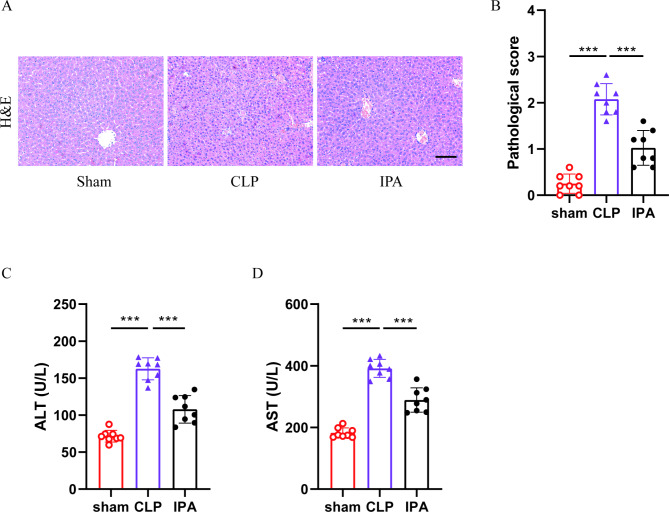
**IPA alleviates liver injury in septic mice.** (**A**-**B**) The H&E staining (**A**) and pathological scoring (**B**) of liver tissue sections (n = 8), the scale bar is 100 μm. (**C**) The plasma ALT levels in mice (n = 8). (**D**) The plasma AST levels in mice (n = 8). The results are expressed as the mean ± SD, “*” indicates *p* < 0.05, “**” indicates *p* < 0.01, “***” indicates *p* < 0.001, *p* were determined by ANOVA and post-hoc test

Then the effect of IPA on liver injury in septic mice was observed. Compared with the sham group, CLP caused significant H&E pathological damage in liver tissue (Fig. 4-A-B) and increases in plasma ALT (Fig. 4C) and AST (Fig. 4D) levels. While IPA pretreatment significantly inhibited CLP-induced liver injury. These results suggested that IPA has a significant effect on reducing sepsis-induced liver injury.

### IPA alleviates liver injury in septic mice by activating PXR

**Fig. 5:**
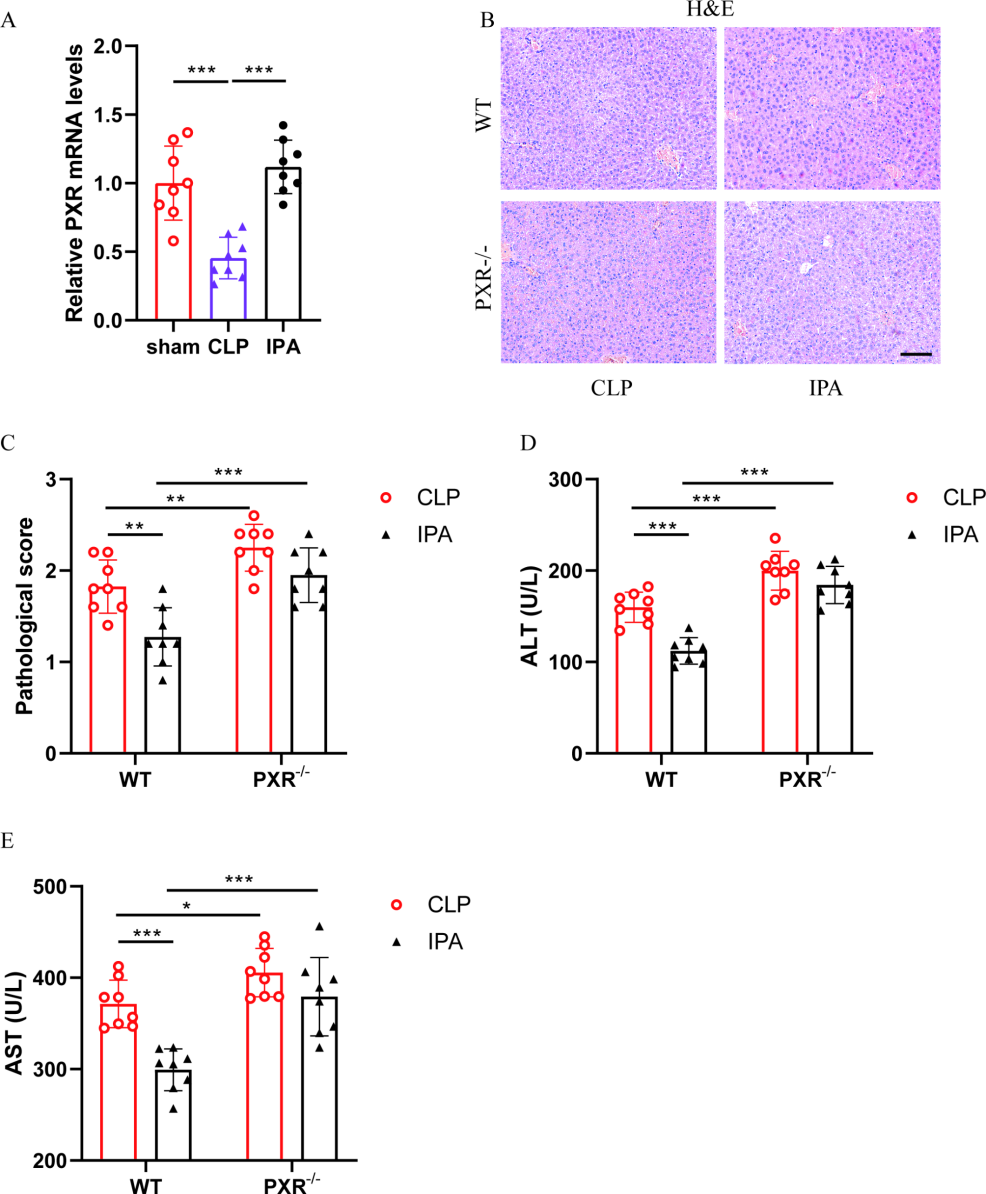
**IPA alleviates liver injury in septic mice by activating PXR.** (**A**) The relative mRNA level of PXR (n = 8). (**B**-**C**) The H&E staining (**B**) and pathological scoring (**C**) of liver tissue sections (n = 8), the scale bar is 100 μm. (**D**) The plasma ALT levels in mice (n = 8). (**E**) The plasma AST levels in mice (n = 8). The results are expressed as the mean ± SD, “*” indicates *p* < 0.05, “**” indicates *p* < 0.01, “***” indicates *p* < 0.001, *p* were determined by ANOVA and post-hoc test

As shown in Fig. 5A, IPA pretreatment obviously reversed the tendency that CLP inhibited PXR mRNA expression in liver tissue. To investigate the role of PXR in IPA attenuating sepsis-induced liver injury, PXR knockout mice (PXR^-/-^) were used together with wild-type mice (WT) to establish a CLP model. IPA pretreatment significantly reduced the H&E pathological damage of liver tissue (Fig. 5B-C) and the levels of ALT (Fig. 5D) and AST (Fig. 5E) in plasma of WT septic mice, but not found in PXR^-/-^ septic mice. Furthermore, the H&E pathological damage of liver tissue (Fig. 5B-C) and the levels of ALT (Fig. 5D) and AST (Fig. 5E) in plasma of WT mice were all lower than that in the PXR^-/-^ mice. These results suggested that knockout of PXR abolished the protective effect of IPA against sepsis-induced liver injury.

## Discussion

In this study, we found that the lower IPA levels correlate with more severe SALI. Furthermore, IPA alleviates injury in septic mice, especially SALI, suggesting that IPA is a potential drug for the prevention of sepsis and SALI. In addition, we demonstrated that IPA alleviated SALI by promoting PXR expression, suggesting that PXR signaling is a potential new target for the prevention and treatment of SALI and revealing a new mechanism of SALI.

It has been found that regulating intestinal flora can reduce SALI (Sun et al. 2020; Liang et al. 2022; Gong et al. 2019; Chancharoenthana et al. 2022; Hiengrach et al. 2022). In this study, we reveal that IPA is a key gut microbiota metabolite affecting SALI, suggesting that IPA is a potential therapeutic agent for the prevention of sepsis and SALI.

It has been confirmed that PXR activation within 1 week after liver transplantation is an indicator of patient prognosis. Furthermore, PXR activation has been confirmed to have a protective effect on cholestatic liver injury (Zeng et al. 2017) and drug-induced liver injury (Zhang et al. 2019). However, the role of PXR in SALI has not been reported. In this study, we demonstrated that the protective effect of IPA against liver injury in septic mice was dependent on the activation of PXR, suggesting that PXR signaling is a potential new target for the prevention and treatment of SALI and revealing a new mechanism of SALI. IPA pretreatment unregulated PXR mRNA expression in liver, and PXR knockout increases pathological scoring of liver tissue in both CLP and IPA treatment groups. However, there is still an observable although not significant effect of the IPA treatment in PXR^-/-^ mice. Together, the results indicate that intact PXR signalling is beneficial to counteract liver dysfunction or injury. It has been demonstrated that PXR regulates gene expression at the translational level and has an extensive protein-protein interaction network through which it participates in cross-signaling pathways. In addition, PXR’s potential role in SALI was highlighted by its interaction with some key proteins in DNA damage pathways, such as p53 and Tip60. Understanding how PXR maintains genomic stability will lead to a better understanding of the underlying mechanism of PXR in SALI.

There are limitations in this study. This study did not reveal the intestinal flora (bacteria, viruses, fungi, and archaea) associated with SALI, and the intestinal bacteria that produce IPA and the changes of these IPA-producing bacteria in sepsis and SALI are unknown. The role of IPA and PXR in SALI remains to be verified in clinical trials. In order to avoid the interference of gender differences on the intestinal flora and different physiological cycles of female mice to the experimental results, only male mice were selected for the experimental mice. Furthermore, we did not observe the regulatory effect of PXR on other genes or proteins in SALI. Knockout of PXR gene does effectively abolish the protective effect of IPA on SALI, but the underlying mechanism of how PXR regulates SALI remains unclear, which is also the focus of our next study. In addition, this study did not explore the optimal dose or dose range of IPA for SALI protection.

## Conclusion

IPA alleviates SALI by activating PXR, which reveals a new mechanism of SALI, and provides potentially effective drugs and targets for the prevention of SALI.

## Electronic supplementary material

Below is the link to the electronic supplementary material.

Supplementary Material 1

Supplementary Material 2

## Data Availability

The datasets used and/or analyzed during the current study are available from the corresponding author on reasonable request.

## Data Availability

The authors confirm that the data supporting the findings of this study are available within the article.

## List of Abbreviations

SALI: Sepsis-associated acute liver injury
SNALI: Sepsis non-acute liver injury
IPA: Indole-3-propionic Acid
PXR: Pregnane X receptor
AST: Aspartate aminotransferase
ALT: Alanine aminotransferase
GGT: γ-glutamyl transpeptidase
MSS: Mouse sepsis injury score
H&E: Hematoxylin and eosin
IQR: Interquartile range
ABX: Antibiotics

## References

[CR26] Adelman MW (2020). The gut microbiome’s role in the development, maintenance, and outcomes of sepsis. Critical care (London, England).

[CR25] Agudelo-Ochoa GM V-DB, Giraldo-Giraldo NA, Jaillier-Ramírez AM, Giraldo-Villa A, Acevedo-Castaño I, Yepes-Molina MA, Barbosa-Barbosa J, Benítez-Paéz A (2020). Gut microbiota profiles in critically ill patients, potential biomarkers and risk variables for sepsis. Gut Microbes.

[CR9] Ahmed BA (2021). Lower brown adipose tissue activity is associated with non-alcoholic fatty liver disease but not changes in the gut microbiota. Cell reports. Medicine.

[CR33] Barretto SA (2021). The pregnane X receptor drives sexually dimorphic hepatic changes in lipid and xenobiotic metabolism in response to gut microbiota in mice. Microbiome.

[CR11] Bulfoni MPR, Dalla E, Cesselli D, Hidaka M, Di Loreto C, Eguchi S, Baccarani U (2021). miRNA expression profiles in liver grafts of HCV and HIV/HCV-infected recipients, 6 months after liver transplantation. J Med Virol.

[CR44] Chancharoenthana W (2022). Critical roles of sepsis-reshaped fecal virota in attenuating sepsis severity. Front Immunol.

[CR22] Deng F (2021). Gut Microbial Metabolite Pravastatin attenuates intestinal Ischemia/Reperfusion Injury through promoting IL-13 release from type II innate lymphoid cells via IL-33/ST2 signaling. Front Immunol.

[CR23] Deng F (2021). The gut microbiota metabolite capsiate promotes Gpx4 expression by activating TRPV1 to inhibit intestinal ischemia reperfusion-induced ferroptosis. Gut Microbes.

[CR2] Deng F, et al (2022a) Gut microbiota dysbiosis is associated with sepsis-induced cardiomyopathy in patients: a case-control study. Journal of medical virology: e28267. 10.1002/jmv.28267 36319439

[CR20] Deng F (2022). The role of intestinal microbiota and its metabolites in intestinal and extraintestinal organ injury induced by intestinal ischemia reperfusion injury. International journal of biological sciences.

[CR21] Deng F (2022). Propionate alleviates myocardial ischemia-reperfusion injury aggravated by angiotensin II dependent on caveolin-1/ACE2 axis through GPR41. International journal of biological sciences.

[CR28] Deng F (2023). Gut microbe-derived milnacipran enhances tolerance to gut ischemia/reperfusion injury. Cell reports. Medicine.

[CR30] Fang H (2022). Indole-3-Propionic Acid as a potential therapeutic Agent for Sepsis-Induced Gut Microbiota Disturbance. Microbiol Spectr.

[CR32] Fang H (2022). Sepsis-Induced Gut Dysbiosis mediates the susceptibility to Sepsis-Associated Encephalopathy in mice. mSystems.

[CR36] Flannigan KL, *et al* (2022) The Pregnane X Receptor and Indole-3-Propionic Acid Shape the Intestinal Mesenchyme to Restrain Inflammation and Fibrosis. *Cellular and molecular gastroenterology and hepatology*. 10.1016/j.jcmgh.2022.10.014 PMC9883297 36309199

[CR7] Gao WZY, Ye J, Chu H (2021). Gut non-bacterial microbiota contributing to alcohol-associated liver disease. Gut Microbes.

[CR8] Ghodsian N (2021). Electronic health record-based genome-wide meta-analysis provides insights on the genetic architecture of non-alcoholic fatty liver disease. Cell reports. Medicine.

[CR43] Gong S (2019). Intestinal microbiota mediates the susceptibility to Polymicrobial Sepsis-Induced Liver Injury by Granisetron Generation in mice. Hepatology (Baltimore, Md.).

[CR29] Haak BW, Wiersinga WJ (2017). The role of the gut microbiota in sepsis. The lancet. Gastroenterology & hepatology.

[CR45] Hiengrach P, Panpetch W, Chindamporn A, Leelahavanichkul A (2022). Macrophage depletion alters bacterial gut microbiota partly through fungal overgrowth in feces that worsens cecal ligation and puncture sepsis mice. Scientific reports.

[CR31] Huang ZB (2022). Gut microbiota-derived indole 3-propionic acid partially activates aryl hydrocarbon receptor to promote macrophage phagocytosis and attenuate septic injury. Frontiers in cellular and infection microbiology.

[CR34] Jiang Y (2019). Pregnane X receptor regulates liver size and liver cell fate by Yes-Associated protein activation in mice. Hepatology (Baltimore, Md.).

[CR19] Khan A (2021). Understanding the Effects of Gut Microbiota Dysbiosis on nonalcoholic fatty liver Disease and the possible Probiotics Role: recent updates. International journal of biological sciences.

[CR37] Kobashi H, Toshimori J, Yamamoto K (2013). Sepsis-associated liver injury: incidence, classification and the clinical significance. Hepatology research: the official journal of the Japan Society of Hepatology.

[CR15] Li Z (2022). Gut microbiota modulate radiotherapy-associated antitumor immune responses against hepatocellular carcinoma Via STING signaling. Gut Microbes.

[CR42] Liang H (2022). Metformin attenuated sepsis-related liver injury by modulating gut microbiota. Emerging microbes & infections.

[CR5] Mayneris-Perxachs J (2021). Iron status influences non-alcoholic fatty liver disease in obesity through the gut microbiome. Microbiome.

[CR27] Miller WD, Keskey R, Alverdy JC (2021). Sepsis and the Microbiome: a vicious cycle. The Journal of infectious diseases.

[CR6] Rao Y (2021). Gut Akkermansia muciniphila ameliorates metabolic dysfunction-associated fatty liver disease by regulating the metabolism of L-aspartate via gut-liver axis. Gut Microbes.

[CR38] Rittirsch D, Huber-Lang MS, Flierl MA, Ward PA (2009). Immunodesign of experimental sepsis by cecal ligation and puncture. Nature protocols.

[CR35] Sachdeva K, Yan B, Chichester CO (2003). Lipopolysaccharide and cecal ligation/puncture differentially affect the subcellular distribution of the pregnane X receptor but consistently cause suppression of its target genes CYP3A. Shock (Augusta, Ga.).

[CR16] Sangineto M (2022). Recovery of Bacteroides thetaiotaomicron ameliorates hepatic steatosis in experimental alcohol-related liver disease. Gut Microbes.

[CR3] Shi YZY, Xiong Y, Zhang S, Song M, An X, Liu C, Zhang W, Chen S (2021). Host gasdermin D restrains systemic endotoxemia by capturing Proteobacteria in the colon of high-fat diet-feeding mice. Gut Microbes.

[CR39] Shrum B (2014). A robust scoring system to evaluate sepsis severity in an animal model. BMC Res. Notes.

[CR13] Sun J (2020). Gut-liver crosstalk in sepsis-induced liver injury. Critical care (London, England).

[CR4] Sun Z (2021). 17β-Estradiol promotes LC3B-associated phagocytosis in trained immunity of female mice against sepsis. International journal of biological sciences.

[CR41] Suzuki S, Toledo-Pereyra LH, Rodriguez FJ, Cejalvo D (1993). Neutrophil infiltration as an important factor in liver ischemia and reperfusion injury. Modulating effects of FK506 and cyclosporine. Transplantation.

[CR10] Wang YLX, Chen Q, Jiao F, Shi C, Pei M, Wang L, Gong Z (2021). The relationship between liver pathological inflammation degree and pyroptosis in chronic hepatitis B patients. J Med Virol.

[CR17] Wang F (2021). Regorafenib plus toripalimab in patients with metastatic colorectal cancer: a phase Ib/II clinical trial and gut microbiome analysis. Cell reports. Medicine.

[CR14] Wang H (2022). Bacteroides acidifaciens in the gut plays a protective role against CD95-mediated liver injury. Gut Microbes.

[CR12] Woźnica EA, Inglot M, Woźnica RK, Łysenko L (2018). Liver dysfunction in sepsis. Advances in clinical and experimental medicine: official organ Wroclaw Medical University.

[CR18] Xiang H (2022). Dynamics of the gut-liver axis in rats with varying fibrosis severity. International journal of biological sciences.

[CR40] Xue H (2022). Gut Microbially Produced Indole-3-Propionic Acid inhibits atherosclerosis by promoting reverse cholesterol transport and its Deficiency is causally related to atherosclerotic Cardiovascular Disease. Circulation research.

[CR46] Zeng H (2017). Schisandrol B protects against cholestatic liver injury through pregnane X receptors. British journal of pharmacology.

[CR47] Zhang J (2019). Genetic polymorphisms in PXR and NF-κB1 influence susceptibility to anti-tuberculosis drug-induced liver injury. PloS one.

[CR24] Zhang XY (2020). Phlorizin ameliorates obesity-associated endotoxemia and insulin resistance in high-fat diet-fed mice by targeting the gut microbiota and intestinal barrier integrity. Gut Microbes.

[CR1] Zheng X, Chen W, Gong F, Chen Y, Chen E (2021). The role and mechanism of pyroptosis and potential therapeutic targets in Sepsis: a review. Front Immunol.

