# Supplementary figures and images for: Indole-3-propionic acid alleviates sepsis-associated acute liver injury by activating pregnane X receptor
Source: Mol Med. 2023 May 19;29:65. doi: 10.1186/s10020-023-00658-x (PMC10197241; doi:10.1186/s10020-023-00658-x)

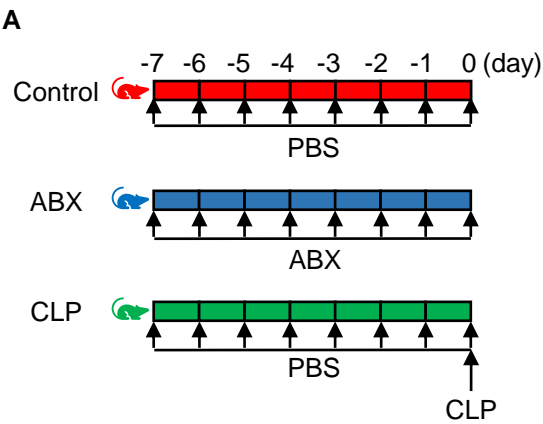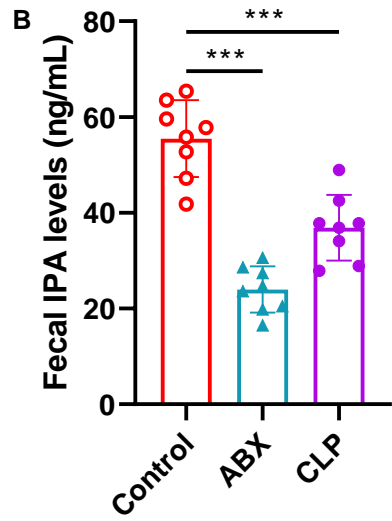

Supplement: Supplementary file 1 — Supplementary Material 1 [file 10020_2023_658_MOESM1_ESM.pdf]
