# Supplementary material for: Indole-3-propionic acid alleviates sepsis-associated acute liver injury by activating pregnane X receptor
Source: Mol Med. 2023 May 19;29:65. doi: 10.1186/s10020-023-00658-x (PMC10197241; doi:10.1186/s10020-023-00658-x)
Supplement: Supplementary file 2 — Supplementary Material 2 [file 10020_2023_658_MOESM2_ESM.docx]

**Cover letter**

[01/04/2023]

Professor Betty Diamond

### Editor-in-Chief

### Molecular Medicine

Manuscript No. MOME-D-23-00037

Tltle: " Indole-3-propionic Acid, a metabolite of gut microbiota, alleviates sepsis-associated acute liver injury by activating pregnane X receptor"

Dear Editor, Dear reviewers

Thank you for giving us the opportunity to submit a revised manuscript “Indole-3-propionic Acid, a metabolite of gut microbiota, alleviates sepsis-associated acute liver injury by activating pregnane X receptor (manuscript No. MOME-D-23-00037)” in the Journal of Molecular Medicine. We appreciate the time and effort that you and the reviewers dedicated to providing feedback on our manuscript and are grateful for the insightful comments on and valuable improvements to our paper.

According to the reviewers’ comments, we have revised the manuscript extensively. If there are any other modifications we could make, we would like very much to modify them and we really appreciate your help. We hope that our manuscript could be considered for publication in Molecular Medicine. Thank you very much for your help.

Thank you again for your positive comments on our manuscript. Molecular Medicine is an influential journal which aims to improve our understanding of translational and clinical biomedical sciences that inform and influence human health and medicine. From all the papers published in your journal, readers have been learning a lot. Hopefully, we could have our article been considered of publication in your journal. Should there been any other corrections we could make, please feel free to contact us.

Based on these comments and suggestions, we have made careful modifications to the original manuscript, and carefully proof-read the manuscript to minimize typographical and grammatical errors. We believe that the manuscript has been greatly improved and hope it has reached your magazine’ s standard.

**Response to Reviewers’ Comments**

**Reviewers' comments:**
***Reviewer #1***

***Reviewer #1:*** *The authors have significantly improved the revised manuscript and addressed my comments.
However, I still see minor points that need to be raised:*

*1. The authors make the claim repeatedly that they show that "IPA is a metabolite of gut microbiota" (e.g. line 35). This is no novel finding and should not be presented as such.*
**Response:** We are very grateful for Reviewer #1's valuable comments. Accordingly, we have corrected the description.

*2. Figure 3C shows significance, however the comparisons are not deductible from the plot.*

**Response:** We are very grateful for Reviewer #1's valuable comments. Accordingly, we have corrected.

*3. In Figure 3D and E, the authors have performed qPCR of transcripts encoding occluding and ZO-1 for samples obtained from the sham, CLP and IPA group and say that "IPA treatment significantly promoted the expression of [...] Occludin and and ZO-1 in intestinal tissue of sepsis mice". While that statement is correct, it makes me wonder if IPA treatment of non-septic mice would also induce increased intestinal barrier integrity? Could the authors perform a similar comparison between mice treated with and without IPA and detect transcriptional upregulation of occluding, OZ-1 and the potential underlying regulator PXR, in the absence of a sepsis model? This result would significantly support their main conclusion.*

**Response:** We are very grateful for Reviewer #1's valuable comments. Accordingly, we have added the data that the mRNA levels of Occludin, ZO-1, PXR between mice treated with and without IPA. As shown below, IPA treatment significantly increased the mRNA expression of the intestinal barrier tight junction markers Occludin and ZO-1 and IPA in liver tissue. Therefore, the result significantly support our main conclusion. Since it was not continuous with the main figure results, we did not put the results of these three mRNAs into the main figure. Thanks again.

*4. The methods section has been extensively edited and now includes more information on animal handling, as well as qPCR and IPA quantification. However, some parts of the section, e.g. the mouse sepsis model description, read like a bullet point protocol (lines 116-118) and could be improved.*

**Response:** We are very grateful for Reviewer #1's valuable comments. Accordingly, we have corrected.

Finally, thanks again to all editors and reviewers for their comments, which were extremely effective in enhancing our manuscript. We hope the changes we have made address all your concerns about this article. We would be more than happy to make further changes to improve the paper and/or facilitate its successful publication.
